# Supplementary material for: Assessment of the Degradation Potential and Genomic Insights towards Phenanthrene by Dietzia psychralcaliphila JI1D
Source: Microorganisms. 2021 Jun 19;9(6):1327. doi: 10.3390/microorganisms9061327 (PMC8235520; doi:10.3390/microorganisms9061327)
Supplement: Supplementary file 1 [file microorganisms-09-01327-s001.zip › microorganisms-1244174-supplementary.pdf]

microorganisms

Supplementary Material

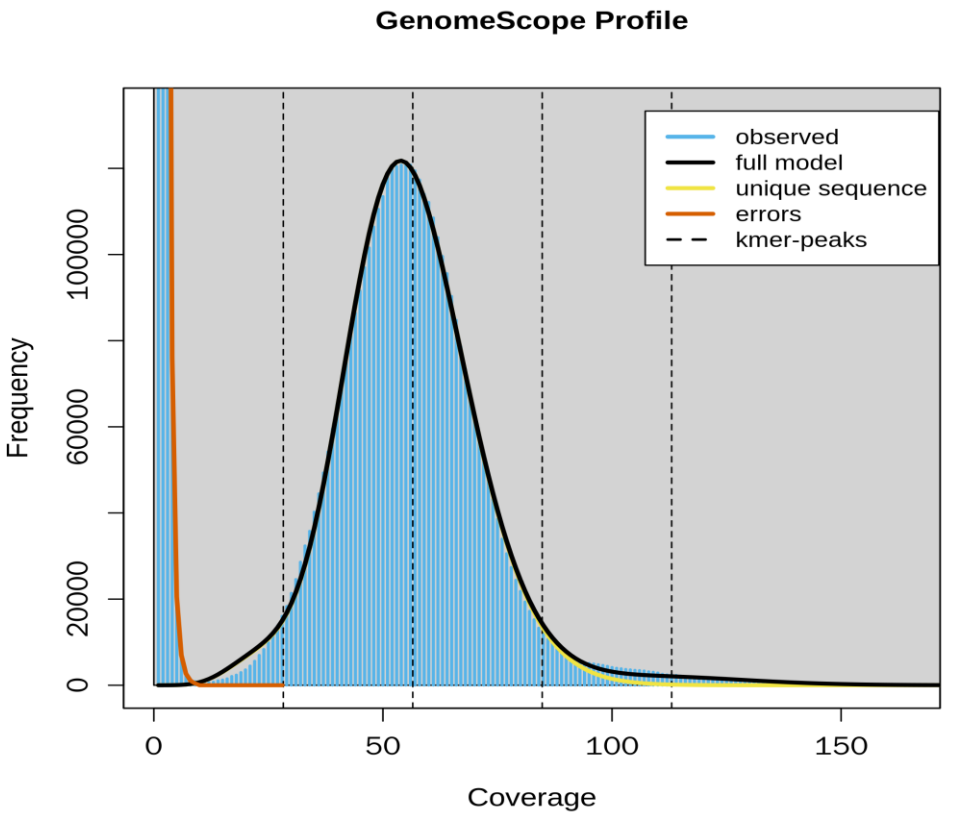

Figure S1. Genome scope profile of k-mer analysis of the genome.

Table 1. k-mer results.

Content	K-mer coverage	Heterozygosity	Genome length	Genome Repeat length
2lmer	56.5	0.11	4,269,056	242,866

Table S2. BUSCO assessments data.

Status	# of BUSCOs	Percentage (%)
Complete and single-copy BUSCOs (S)	141	95.27
Complete and duplicated BUSCOs (D)	0	0
Fragmented BUSCOs (F)	1	0.68
Missing BUSCOs (M)	6	4.05
Total BUSCO groups searched	148	100

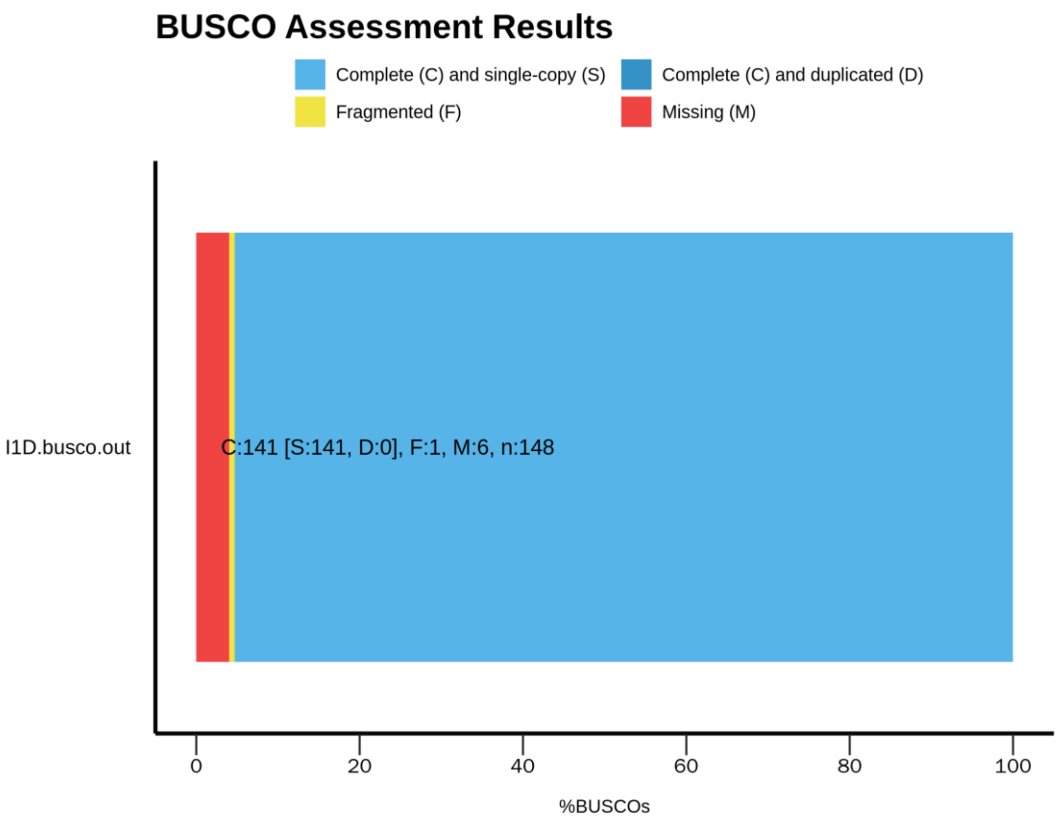

Figure S2. BUSCO result plot of the genome.

Table S3. Overall mapping statistics.

Library name	Total reads	Mapped reads	Mapped reads (%)	Coverage (%)	Depth
JI1D	2,071,944	2,065,009	99.67	100.00	70.94

Table S4. Number of A, T, G, C, N nucleotides present in whole genome sequence.

Number of A	Number of T	Number of G	Number of C	Number of N	GC contents
644,045	648,162	1,477,068	1,447,109	96	69.3%

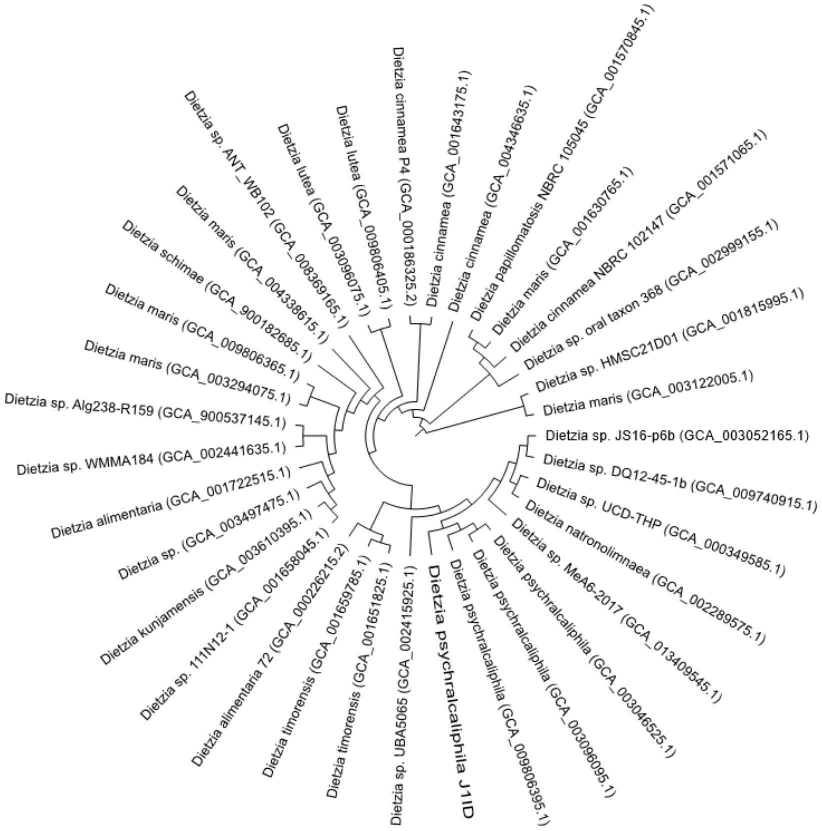

Figure S3. SCG analysis of the strain *D. psychralcaliphila* JI1D.

Table S5. Contents of COGs present in *D. psychralcaliphila* JI1D.

COG Categories	Number of COGs	% COGs
(J) Translation, ribosomal structure, and biogenesis	173	4.596174
(A) RNA processing and modification	2	0.053135
(K) Transcription	329	8.740701
(L) Replication, recombination, and repair	236	6.269926
(B) Chromatin structure and dynamics	2	0.053135
(D) Cell cycle control, cell division, chromosome partitioning	54	1.434644
(V) Defense mechanisms	73	1.939426
(T) Signal transduction mechanisms	116	3.081828
(M) Cell wall/membrane/envelope biogenesis	161	4.277365
(N) Cell motility	18	0.478215
(Z) Cytoskeleton	1	0.026567
(U) Intracellular trafficking, secretion, and vesicular transport	54	1.434644
(O) Posttranslational modification, protein turnover, chaperones	104	2.763018
(C) Energy production and conversion	257	6.827843
(G) Carbohydrate transport and metabolism	164	4.357067
(E) Amino acid transport and metabolism	310	8.235919
(F) Nucleotide transport and metabolism	79	2.098831
(H) Coenzyme transport and metabolism	146	3.878852
(I) Lipid transport and metabolism	357	9.484591
(P) Inorganic ion transport and metabolism	264	7.013815
(Q) Secondary metabolites biosynthesis, transport and catabolism	228	6.057386
(S) Function unknown	636	16.89692

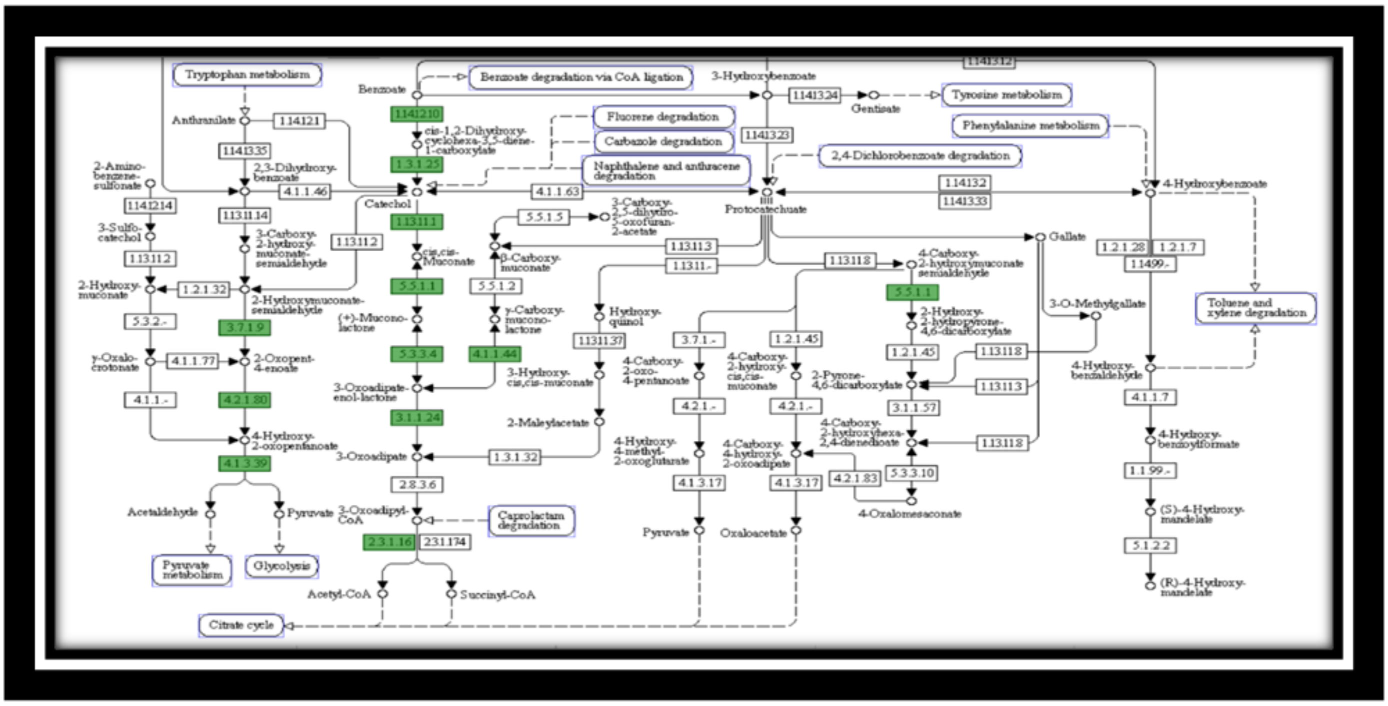

Figure S4. The complete genetic machinery present in the process of benzoate degradation obtained from RAST server. The genes encoded in the green colour boxes are found present in the whole genome analysis.

Table S6. Genes involved in aromatic compounds degradation.

S.No	Gene Name	EC number	Name
**n-Phenylalkanoic acid degradation**			
1	I1D_rast.CDS.1258	2.3.1.16	3-ketoacyl-CoA thiolase
2	I1d_rast.CDS.797	4.2.1.17	Enoyl-CoA hydratase
3	I1d_rast.CDS.381	6.2.1.3	Long chain fatty acid CoA ligase
4	I1D_rast.CDS.511	1.1.1.35	3-hydroxyacyl-CoA dehydrogenase
5	I1D.rast.DS.3324	1.14.13.-	Vanilate O-demethyalse oxidoreductase
**Benzoate Degradation**			
6	I1D.rast.CDS.2910	1.3.1.25	1,2-dihydroxycyclohexa-3,5-diene-1-carboxylate dehydrogenase
7	I1D.rast.CDS.1302	1.14.12.10	Benzoate 1,2-dioxygenase alpha subunit
8	IlD.rast.CDS.1303	1.14.12.10	Benzoate 1,2-dioxygenase beta subunit
9	I1D.rast.CDS.2906		Benzoate transporter protein
10	I1D.rast.CDS.2909	BenK	Benzoate MFS transporter
11	I1D.rast.CDS.2394	3.7.1.-	2-hydroxy-6-oxo-6-phenylhexa-2,4-dienoate hydrolase
12	I1D.rast.CDS.2140	1.2.1.10	Acetaldehyde dehydrogenase
13	I1D.rast.CDS.216	3.1.1.24	Beta-ketoadipate enol-lactone hydrolase
**Quinate degradation**			
14	I1D.rast.CDS.175	4.2.1.10	3-dehydroquinate dehydratase
15	I1D.rast.CDS.1713	1.14.13.-	Baeyer Villiger monooxygenase
16	I1D.rast.CDS.1297	4.2.-. -	2-oxo-hepta-3-ene-1,7-dioic acid hydrates
17	I1D.rast.CDS.1616	4.2.1.18	Methylglutaconyl-CoA hydratase
18	I1D.rast.CDS.955	2.7.1.71	Shikimate kinase I
**Metabolism of Central aromatic compounds**			
19	I1D.rast.CDS.1296	4.1.2.-	2,4-dihydroxyhept-2-ene-1,7-dioic acid aldolase
20	I1D.rast.CDS.1298	3.7.1.9	2-hydroxymuconoic semialdehyde hydrolase

Catechol branch of beta ketoadipate pathway			
21	I1D.rast.CDS.216	3.1.1.24	Beta-ketoadipate enol-lactone hydrolase
22	I1D.rast.CDS.2914	1.13.11.1	Catechol 1,2-dioxygenase
23	I1D.rast.CDS.2915	5.5.1.1	Muconate cycloisomerase
24	I1D.rast.CDS.2916	5.3.3.4	Muconolactone isomerase
25	I1D.rast.CDS.1507	2.8.3.5	Succinyl-CoA:3-ketoacid coenzyme A transferase subunit A
26	I1D.rast.CDS.1506	2.8.3.5	Succinyl-CoA:3-ketoacid coenzyme A transferase subunit B
**Homogentisate pathway**			
27	I1D.rast.CDS.784	IcI R family	Transcriptional regulator
**Protocatechuate branch of beta-ketoadipate pathway**			
28	I1D.rast.CDS.75	4.1.1.44	4-carboxymuconolactone decarboxylase
29	I1D.rast.CDS.2918	PcaR	Pca regulon regulatory protein

Table S7. The legend for Figure 10.

**Compounds**	**Genes involved**	**Compound name**
A	NA	Phenanthrene
B (I)	DOCFNAII_02907, DOCFNAII_01294, DOCFNAII_01236, DOCFNAII_02906, DOCFNAII_01295	Cis-3,4-Dihydroxy-3,4-dihydrophenanthrene
C (II)	DOCFNAII_00095, DOCFNAII_00124	3,4-Dihydroxyphenanthrene
D (III)	DOCFNAII_02135	2-Hydroxy-2H-benzo[h]chromene-2-carboxylate
E (IV)	DOCFNAII_00917, DOCFNAII_03808	Cis-4-(1′-Hydroxy-naphth-2′yl)-2-oxobut-3-enoate
F (V)	DOCFNAII_03745	1-Hydroxy-2-naphthaldehyde
G (VI)	DOCFNAII_03312	1-Hydroxy-2-naphthoate
H (VII)	DOCFNAII_03142, DOCFNAII_02814	Cis-2′-Carboxybenzalpyruvate
I (VIII)	DOCFNAII_00143, DOCFNAII_03745	2-Carboxybenzaldehyde
J (IX)	DOCFNAII_01122, DOCFNAII_01146	Phthalate
K (X)	DOCFNAII_02907	Phthalate-4,5-cis-dihydrodiol
L (XI)	DOCFNAII_03730	4,5-Dihydroxyphthalate
M (XII)	DOCFNAII_02859	3,4-Dihydroxybenzoate
N (XIII)	DOCFNAII_02881, DOCFNAII_03605	4-Carboxy-2-hydroxymuconate semialdehyde
O (XIV)	DOCFNAII_02907, DOCFNAII_01294	1,2-Dihydroxynaphthoate
P (XV)	DOCFNAII_03360	Cis-1,2-Dihydroxy-1,2-dihydronaphthalene
Q (XVI)	DOCFNAII_02135	1,2-Dihydroxynaphthalene
R(XVII)	DOCFNAII_00917	2-Hydroxychromene-2-carboxylate
S (XVIII)	DOCFNAII_03745	Trans-o-Hydroxy-benzylidene pyruvate
T (XIX)	DOCFNAII_02167	Salicylaldehyde
U (XX)	DOCFNAII_02881, DOCFNAII_02482	Salicylate
V	NA	Catechol
